# Microbiome composition as a potential predictor of longevity in rabbits

**DOI:** 10.1186/s12711-024-00895-6

**Published:** 2024-04-02

**Authors:** Iliyass Biada, Noelia Ibáñez-Escriche, Agustín Blasco, Cristina Casto-Rebollo, Maria A. Santacreu

**Affiliations:** Institute for Animal Science and Technology, Universitat Politècnica de València, 46022 Valencia, Spain

## Abstract

**Background:**

Longevity and resilience are two fundamental traits for more sustainable livestock production. These traits are closely related, as resilient animals tend to have longer lifespans. An interesting criterion for increasing longevity in rabbit could be based on the information provided by its gut microbiome. The gut microbiome is essential for regulating health and plays crucial roles in the development of the immune system. The aim of this research was to investigate if animals with different longevities have different microbial profiles. We sequenced the 16S rRNA gene from soft faeces from 95 does. First, we compared two maternal rabbit lines with different longevities; a standard longevity maternal line (A) and a maternal line (LP) that was founded based on longevity criteria: females with a minimum of 25 parities with an average prolificacy per parity of 9 or more. Second, we compared the gut microbiota of two groups of animals from line LP with different longevities: females that died/were culled with two parities or less (LLP) and females with more than 15 parities (HLP).

**Results:**

Differences in alpha and beta diversity were observed between lines A and LP, and a partial least square discriminant analysis (PLS-DA) showed a high prediction accuracy (> 91%) of classification of animals to line A versus LP (146 amplicon sequence variants (ASV)). The PLS-DA also showed a high prediction accuracy (> 94%) to classify animals to the LLP and HLP groups (53 ASV). Interestingly, some of the most important taxa identified in the PLS-DA were common to both comparisons (*Akkermansia*, *Christensenellaceae R-7*, *Uncultured* *Eubacteriaceae*, among others) and have been reported to be related to resilience and longevity.

**Conclusions:**

Our results indicate that the first parity gut microbiome profile differs between the two rabbit maternal lines (A and LP) and, to a lesser extent, between animals of line LP with different longevities (LLP and HLP). Several genera were able to discriminate animals from the two lines and animals with different longevities, which shows that the gut microbiome could be used as a predictive factor for longevity, or as a selection criterion for these traits.

**Supplementary Information:**

The online version contains supplementary material available at 10.1186/s12711-024-00895-6.

## Background

Longevity and resilience are different concepts but are highly related. In livestock production, longevity refers to the length of the productive life of a farm animal [1]. Resilience has been defined as the animal's ability to be minimally impacted by disturbances and to quickly regain its production performance after an environmental perturbation [2]. A positive genetic correlation has been reported between longevity and resilience [3–6], which suggests that animals with better resilience tend to have greater longevity. This correlation can be attributed to specific traits that are associated with resilience, such as disease resistance and reproductive performance. These traits can reduce the occurrence of involuntary culling and thereby increase longevity [1].

In rabbits, longevity has a lower economic weight than traits such as litter size and feed efficiency [7]. As a result, longevity has not been included in the breeding goals for rabbits. However, selection that focuses only on increasing productive traits has led to replacement rates of 120% per year [8] and to nearly 50% of all females replaced during their first three parities [9], which is a direct indicator of the degradation of longevity and resilience.

Selection for longevity and resilience is a complicated and slow process. Although longevity has been recorded and evaluated in many studies and species [1, 10, 12], there has been no consensus on how to define the trait and how to analyse the phenotypic data collected for genetic evaluation. As a result, heritability estimates found in the literature are quite heterogenous [10, 11]. In rabbits, the mean heritability is around 0.13, but ranges from 0.02 to 0.24, depending on the model and the definition of the trait [12]. Similarly, studies on resilience are quite heterogeneous and conditioned by the resilience indicator used [13]. Differences between expected patterns and fluctuations in performance (environmental variance) have been described as a promising indicator of resilience [13]. In rabbits, an experiment of divergent selection for environmental variance of litter size showed that animals with a low(er) variance of litter size coped better with environmental disturbances, while those with a high(er) variance were more affected [14].

The gut microbiota influences the host’s resilience and longevity by affecting various physiological functions [15]. Studies in germ-free animals have shown extensive and complex interactions between microbial cells and the host immune system [16]. These interactions are necessary for proper development and maturation of the gut immune system [17]. In addition, the gastrointestinal microbial community acts as an anti-infectious barrier by a mechanism called ‘colonization resistance’, which inhibits adherence and subsequent colonization of pathogens [18, 19]. Through these processes, the gut microbiota could influence longevity and resilience.

Microbial communities can be characterized by amplifying and sequencing the 16S rRNA gene from biological samples (tissue or faecal samples) [20]. Recent advances in metagenomics and gut microbiome studies may allow us to explore novel ways to improve longevity and resilience in rabbit breeding. E.g., gut microbiota profiles could be incorporated into selection criteria for longevity and resilience in rabbits, if they play a role on their phenotypic variation [21, 22]. In addition, if specific taxa within gut microbiota were found to influence longevity and resilience, they could be incorporated into prediction models (similar to genetic markers) or allow development of probiotics to modify the gut microbiota of rabbits.

Therefore, the aim of this study was to compare the gut microbiome profiles of rabbits with different longevities using 16S rRNA gene analysis and to identify discriminating taxa. For this purpose, we compared: (1) the gut microbiota of a high longevity line (LP) with that of a standard longevity maternal line (A); and (2) the gut microbiota of LP line females that died or were culled after two parities or less (LLP) with those with a high longevity, i.e. females with 15 parities or more (HLP).

## Methods

### Animals

In total, 95 females from two rabbit lines (LP and A) currently selected for litter size at weaning were used in this experiment. The 31 females from line A were from the 50th generation of selection and the 64 females from line LP from the 15th generation. Line A is a standard commercial maternal line that was founded by sampling New Zealand White rabbits reared by farmers near Valencia (Spain). Line LP is a robust maternal line that was founded in 2002 by females with a minimum of 25 parities and with an average prolificacy per parity of nine or more, which is the average prolificacy of commercial rabbits in Spain [23]. Since its foundation, the LP line has consistently demonstrated longer longevity compared to line A, with a doe from line A being 1.8 times more likely to die or be culled than a doe from line LP, with a statistical probability of 99% [24]. The females for this experiment were reared in the same conditions and were allowed to reach the maximum number of parities. They were all housed on the farm of the *Universidad Politècnica de Valencia* (UPV) in individual cages (flat‐deck) with an extractable nest box with isolated plastic, and under a photoperiod of 16‐h light: 8‐h dark and controlled temperature and ventilation. Access to the same standard commercial diet was ad libitum for the entire experimental period. The females in this experiment were used for two comparisons: (1) DLINES, which is a comparison between females from line A (n = 31) and line LP (n = 40) with the samples from these females collected during the same trimester of the same year (from 02/07/2018 to 17/09/2018), and (2) DLP, which is a comparison between LP females with different longevities, i.e. LP females with two parities or less during their lifetime (group LLP, n = 19), and LP females who reached 15 parities or more (group HLP, n = 22). The samples of DLP were collected from 30/04/2018 to 06/08/2018 and the HLP and LLP groups were balanced between the two trimesters.

### Sample collection and DNA extraction

During the second week after first parity, daily attempts were made to collect fecal samples from the anus of females by applying gentle pressure to the perianal area. Three separate collections were attempted each day until successfully obtaining a sample. Once the fecal samples were available, they were immediately frozen at − 72 °C until DNA extraction. Bacterial genomic DNA was isolated from the frozen faecal samples using the DNeasy PowerSoil kit (QIAGEN Inc, Hilden, Germany) following the manufacturer's instructions with the following modifications: faecal samples (0.1 g) were disrupted with three 4-mm glass beads in a bead homogeniser (BeadMill 4, ThermoFisher), at maximum speed (6 m/s) for 1 min in the presence of C1 buffer, and incubated at 95 °C for 5 min. These steps were repeated twice. The sample tubes were spun at 10,000*g* for 30 s and the supernatant was transferred to a new tube according to the manufacturer's instructions. In the final step, the DNA was eluted from the column in a 100-µl volume. DNA concentration and purity were estimated first by spectrometry on a Nanodrop ND-1000 and, second, by fluorometry on a Qubit 4 fluorometer (Invitrogen, Thermo Fisher Scientific, Carlsbad, CA, USA) with the dsDNA HS DNA assay kit (Invitrogen).

### PCR amplification, barcoding, and DNA sequencing

Microbial genomic DNA (5 ng/μL in 10 mM Tris pH 8.5) was amplified and purified according to the 16S Metagenomic Sequencing Library Preparation protocol by Illumina. First, regions V3 and V4 of the bacterial 16S rRNA gene were amplified using the following recommended primers (Forward Primer = 50TCGTCGGCAGCGTCAGATGTGTATAAGAGACA GCCTACGGGNGGCWGCAG; Reverse Primer = 50GTCTCGT GGGCTCGGAGATGTGTATAAGAGACAGGACTACHVGGGTATCTAATCC) and the suggested cycling conditions (3 min at 95 °C; 25 cycles of 30 s at 95 °C, 30 s at 55 °C, 30 s at 72 °C; 5 min at 72 °C). After amplification of the 16S rRNA gene, the multiplexing step was performed using the Nextera XT Index Kit (FC-131-2001) by attaching dual indices to both ends of the PCR products. The PCR products (1 μL) were analysed with a Bioanalyzer DNA 1000 chip to verify their size, with the expected size on a Bioanalyzer trace being ~ 550 bp. After size verification, the libraries were sequenced using a 2 × 300 pb paired-end run (MiSeq Reagent kit v3 (MS-102-3003) on a MiSeq Sequencer according to the manufacturer’s instructions (Illumina).

### Bioinformatic analyses

Primary processing was carried out on the raw sequencing reads, starting with a quality control filtering using the fastp program [25] with the following parameters: min_length: 50, trim_qual_right: 30, trim_qual_type: mean and trim_qual_window: 10. Then, the paired-end Miseq Illumina reads (2*300 bp) were processed in R version (4.1.1) [26], using the DADA2 pipeline for Illumina-sequenced fastq [27]. Forward and reverse reads were trimmed to 260 and 240 bp, respectively, to have Q-scores higher than 20. Chimera filtering (using the consensus method) and denoising were applied using the DADA2 pipeline that infers true biological sequences from reads [28]. This resulted in the identification of amplicon sequence variants (ASV), on the basis of which the ASV table was constructed. The taxonomic annotation of ASV was performed using the QIIME2 software version 2021.11 [29], with a Naive Bayes classifier pre-trained on the SILVA v138 database. The ASV that were classified as Eukaryotes or Archaea were removed. For diversity analysis, for each comparison, samples were rarefied to an even sampling depth according to alpha rarefication curves, and then, diversity metrics (alpha and beta) were computed using QIIME2 [29]. Alpha diversity analysis was assessed using the Kruskal–Wallis test based on three alpha diversity indices: Shannon's diversity, observed diversity, and Pielou's evenness [30–32]. Beta diversity differences were assessed with a permutational multivariate analysis of variance (PERMANOVA) using two beta diversity dissimilarity matrices: Bray–Curtis and Jaccard [33, 34]. To rule out differences due to a high degree of dispersion (within-group variance), a permutational analysis of multivariate dispersions (PERMDISP) was performed. Differences in the distribution of alpha and beta diversities between groups were considered significant when the P-value of the test was lower than 0.05. If the results of the statistical tests at the ASV level were significant, alpha and beta diversity tests were performed at the genus level.

### Partial least squares discriminant analyses (PLS-DA)

All statistical analyses of abundance were done in R [26], following Casto-Rebollo et al. [35]. PLS-DA was performed at both the ASV and genus level for each comparison (DLINES and DLP). First, variables (ASV or genera) that were not present in at least 50% of the animals in each line and each group within line LP were removed. A principal component analysis was conducted on each dataset to remove outlier animals, according to the population structure. After exploratory analyses, one was added to all datasets to deal with the remaining zeros. The datasets were transformed by the additive log-ratio (ALR) transformation to consider their compositional nature [36]. The variable (ASV or genus) with the lowest coefficient of variation was used as the reference variable (${x}_{ref}$) and all the other variables as numerator (${x}_{{\text{j}}}$) [36]. ALR transformation was as follows:

1 $$ALR\left({\text{j}}|ref\right)=log\left(\frac{{x}_{{\text{j}}}}{{x}_{ref}}\right)={\text{log}}\left({x}_{{\text{j}}}\right)-{\text{log}}\left({x}_{ref}\right),$$

where the number of total ALR is ${\text{j}}-1$, with ${\text{j}}$ being the total number of variables (ASV or genus) in each dataset. The ASV sequence used as a reference variable for both comparisons (DLINES and DLP) is indicated in Additional file 1: Text S1, while the genus *UCG-005* was used as the reference for both the DLINES and the DLP comparison. Procrustes analysis was performed to verify the quality of each transformation [36] resulting in a Procrustes correlation of ~ 0.99 for all transformations.

PLS-DA was used for the DLINES and DLP comparisons to identify the ASV and genera that can classify rabbits between lines A and LP or between groups of rabbits with different longevities (LLP vs HLP) within line LP. PLS-DA was performed using the ‘mixOmics’ R package [37]. For each comparison, the inputs were a categorical vector **y** that indicates the rabbit population of each sample and an **X** matrix of dimensions n × k, where n is the number of samples and k = j-1, the number of ALR variables (ASV DLINES, k = 259; genus DLINES, k = 61; ASV DLP, k = 349; genus DLP, k = 66). A PLS-DA model with 10 components was fitted for each of the four ALR-transformed datasets. Then, an iterative process was carried out until each model reached the highest classification performance. In each iteration, the optimal number of components for each model was selected considering the balanced error rate (BER) displayed for the Mahalanobis distance, computed by fourfold cross-validation repeated 100 times. The BER is calculated as the average of the errors for each class: BER = 0.5*(FP/(TN + FP) + FN/(FN + TP)), where FP is the false positive count, FN the false negative count, TN the true positive count, and TN the true negative count. Variable selection was performed using variable important prediction (VIP) [38], i.e. the influence of each of the previously selected variable on the model projection and classification. The variables selected for the final PLS-DA were those with a VIP higher than 1 [38]. To check the robustness of the PLS-DA, a confusion matrix and a permutation test were computed using a fourfold cross-validation repeated 10,000 times. The confusion matrix collects the outputs of the classification model and evaluates the classification performance by counting the number of false positives, false negatives, true positives, and true negatives [39]. The permutation test evaluates whether the classification of the individuals to the two groups is significantly better than any other random classification to two arbitrary groups [40].

### Differential abundance analysis

A Bayesian linear model [41] was used for differential abundance analysis. The objective was to determine the relevance of the difference in the ASV and genera selected by the PLS-DA final models between the two rabbit lines A and LP, and between the two groups of LP. The model included the line (A and LP) or LP longevity group (LLP and HLP) as fixed effects for each comparison at the ASV and genus level. The residuals of the model were assumed to follow a normal distribution and the priors for the fixed effects and the residual variance were flat. The Bayesian models were solved by the Gibbs Markov chain Monte Carlo (MCMC) sampling algorithm using 50,000 iterations, a lag of 10 and a burn-in of 1000 iterations. Convergence was checked by the R-hat statistic [42]. Marginal posterior distributions of the differences in the DLINES and the DLP comparisons were computed to estimate the posterior means and the probability of the difference being greater (if the difference is positive) or less (if negative) than 0 (P0). The posterior mean of the differences was expressed in units of standard deviations (SD) for each variable. A Bayesian approximation of the false discovery rate (FDR) was computed by using the cumulative posterior error probability (PEP), similar to the q-value, to establish the threshold for the identification of relevant taxa [35, 40]. The PEP was calculated as (1 − P0)/0.5. We assumed a cumulative PEP of 0.05 as a threshold, meaning that approximately 5% of the taxa that were identified as significant were false positives.

## Results

### Abundance tables and taxonomic annotation

**Fig. 1:**
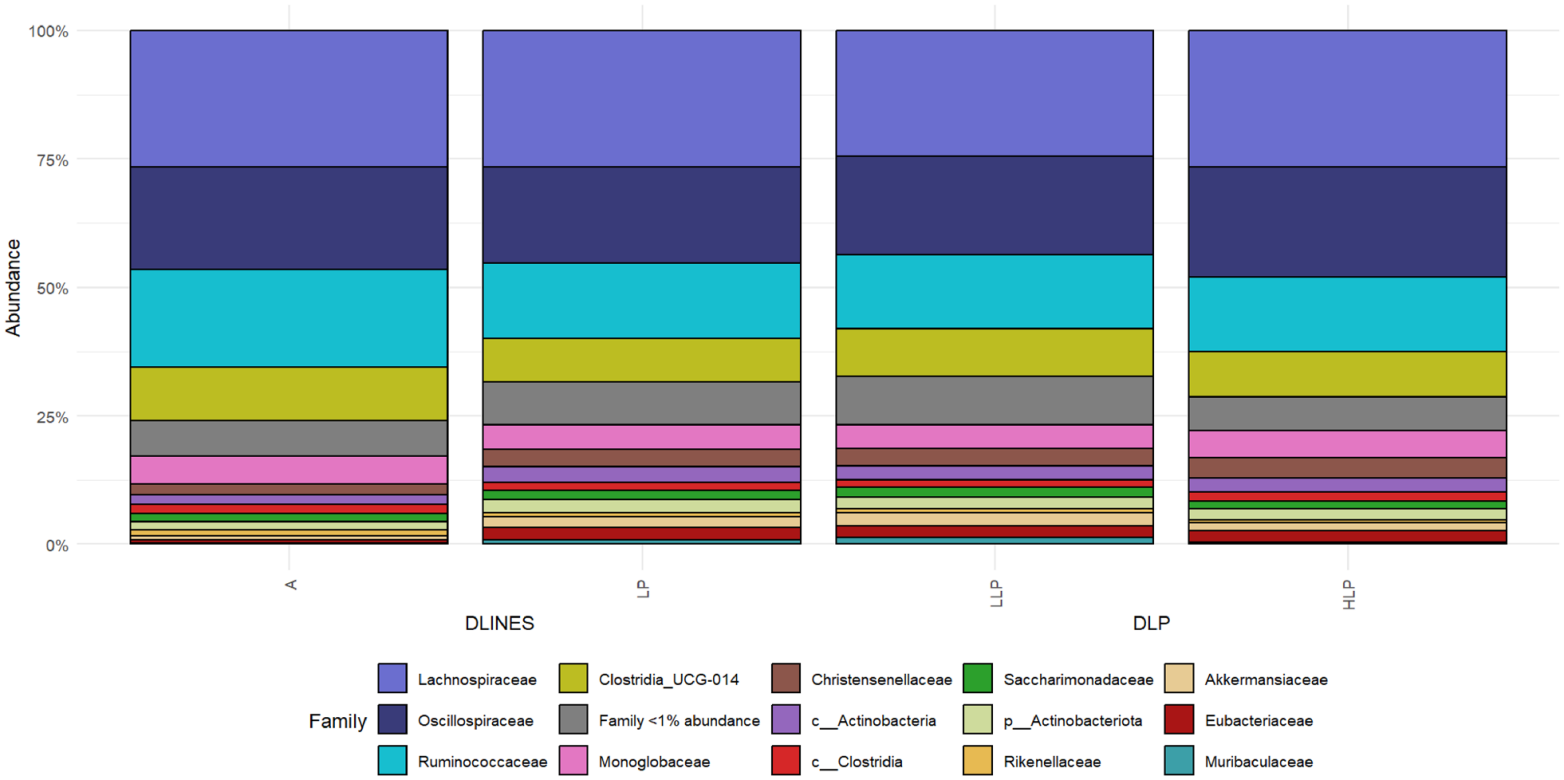
Relative abundance of microbiome families in the A, LP, LLP and HLP groups. DLINES: comparison between A and LP lines; DLP: comparison between LP does with at least two parities and those with at least 15 parities; A line: standard commercial maternal line; LP line: maternal line founded based on longevity criteria; LLP: LP does with two parities or less; and HLP: does with at least 15 parities (HLP)

The final abundance table of all 95 samples used in this experiment contained 6515 ASV and 2,801,584 reads; the ASV sequences are in Additional file 2: Text S2. The mean number of reads per sample was 29,490, with a maximum per sample of 62,439 and a minimum of 10,863. The taxonomic annotation resulted in a total of 11 phyla, 16 classes, 42 orders, 65 families, and 139 genera. Among the 11 phyla detected in all samples, *Firmicutes* were consistently the most abundant phylum, accounting for a relative abundance of 87% (± 0.39). The other most abundant phyla were *Actinobacteria* with 6% (± 0.06) and *Bacteroidota* with 3% (± 0.03). Additional phyla with a sizable abundance included *Verrucomicrobiota* and *Patescibacteria*. At the family level, the most abundant families were *Lachnospiraceae* (26% ± 0.13), *Oscillospiraceae* (20% ± 0.11), *Ruminococcaceae* (15% ± 0.1), and *Clostridia_UCG-014* (9% ± 0.04). Figure 1 illustrates the relative abundance of microbial ASV for the groups used in the two comparisons DLINES (lines A and LP) and DLP (groups LLP and LP).

### Diversity measures

**Fig. 2:**
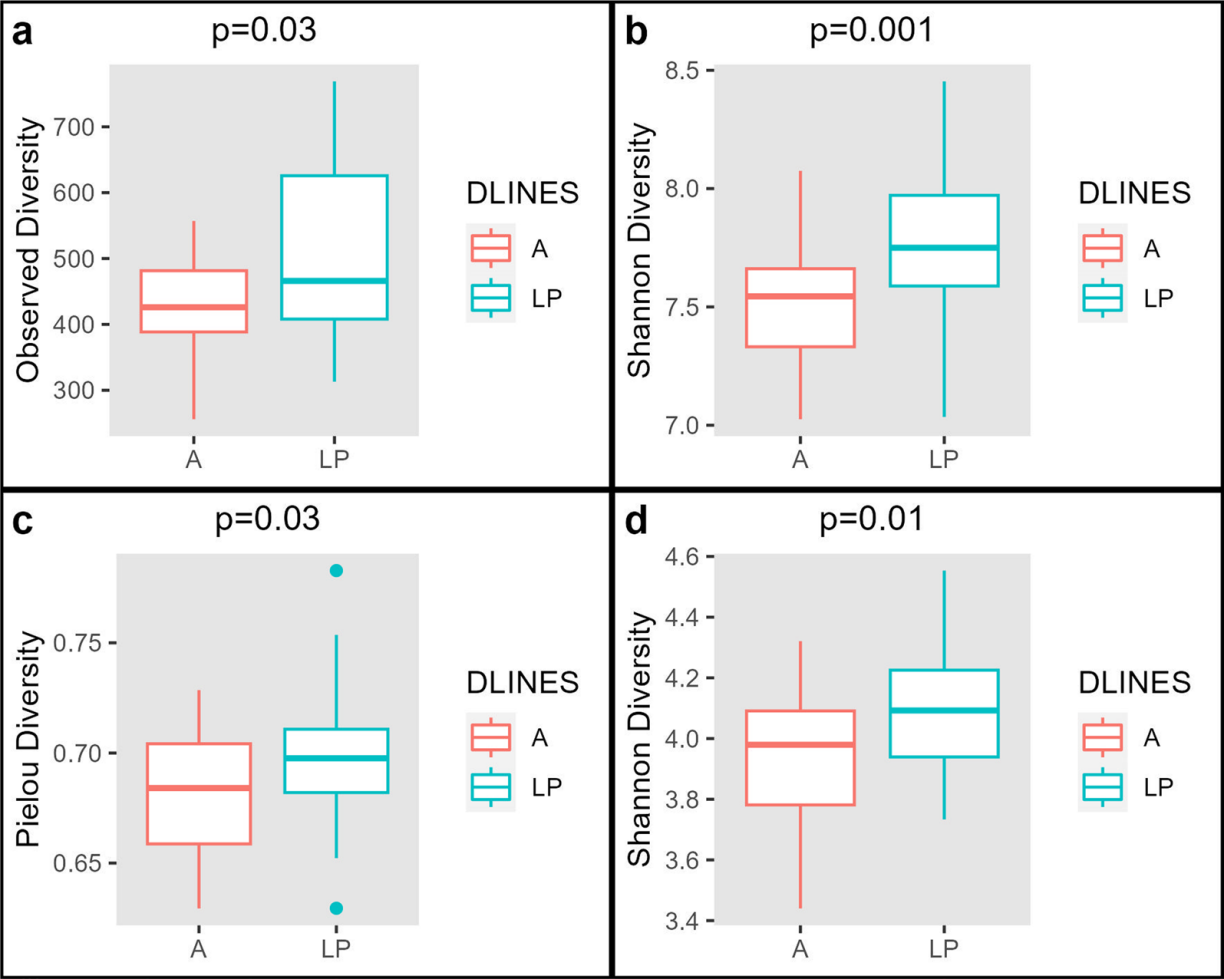
Alpha diversity boxplots of the comparison between lines A and LP. **a** Observed diversity index computed at the amplicon sequence variant (ASV) level for line A (standard commercial maternal line) and line LP (maternal line founded using longevity criteria); **b** Shannon diversity index computed at the ASV level for lines A and LP; **c** Pielou’s evenness computed at the genus level for lines A and LP; **d** Shannon diversity index computed at the genus level for lines A and LP

After rarefaction to 10,226 reads in the DLINES and to 11,108 in the DLP comparison groups, the final dataset had 3887 ASV and 71 samples for the DLINES comparison and 3488 ASV and 41 samples for the DLP comparison. Kruskal–Wallis tests for alpha diversity indices at the ASV level (Fig. 2a and b) showed a significant higher diversity in line LP than in line A for the observed diversity (p-value = 0.03) and Shannon’s (p-value = 0.001) indices. However, alpha diversity using Pielou’s evenness index was not significantly different between lines A and LP (p-value = 0.17) (see Additional file 3: Fig. S1). After rarefaction, the Kruskal–Wallis test results at the genus level showed that Shannon`s (p-value = 0.01) and Pielou’s evenness (p-value = 0.03) indices were both clearly higher for line LP than line A (Fig. 2c and d). These results for alpha-diversity indicate that the microbiota of line LP had higher richness and evenness than that of line A. In the DLP comparison, the alpha-diversity indices showed no difference between the two groups (HLP and LLP) of line LP, (see Additional file 3: Figs. S2, S3 and S4).

The PERMANOVA tests for the DLINES comparison at the ASV level showed differences for both beta-diversity indices, Bray–Curtis (p-value = 0.001) and Jaccard (p-value = 0.001). However, the Bray Curtis index was discarded because the PERMDISP test was significant (p-value = 0.02), which indicates that the differences could be due to large variation within each group. At the genus level, for both indices, the PERMANOVA tests indicated that differences between lines A and LP were significant for both Bray–Curtis (p-value = 0.001) and Jaccard beta (p-value = 0.01) indices. The PERMDISP tests were not significant for both indices, and none of the indices were discarded. Both beta diversity indices were not significantly different for the DLP comparison.

### Partial least squares discriminant analyses

**Fig. 3:**
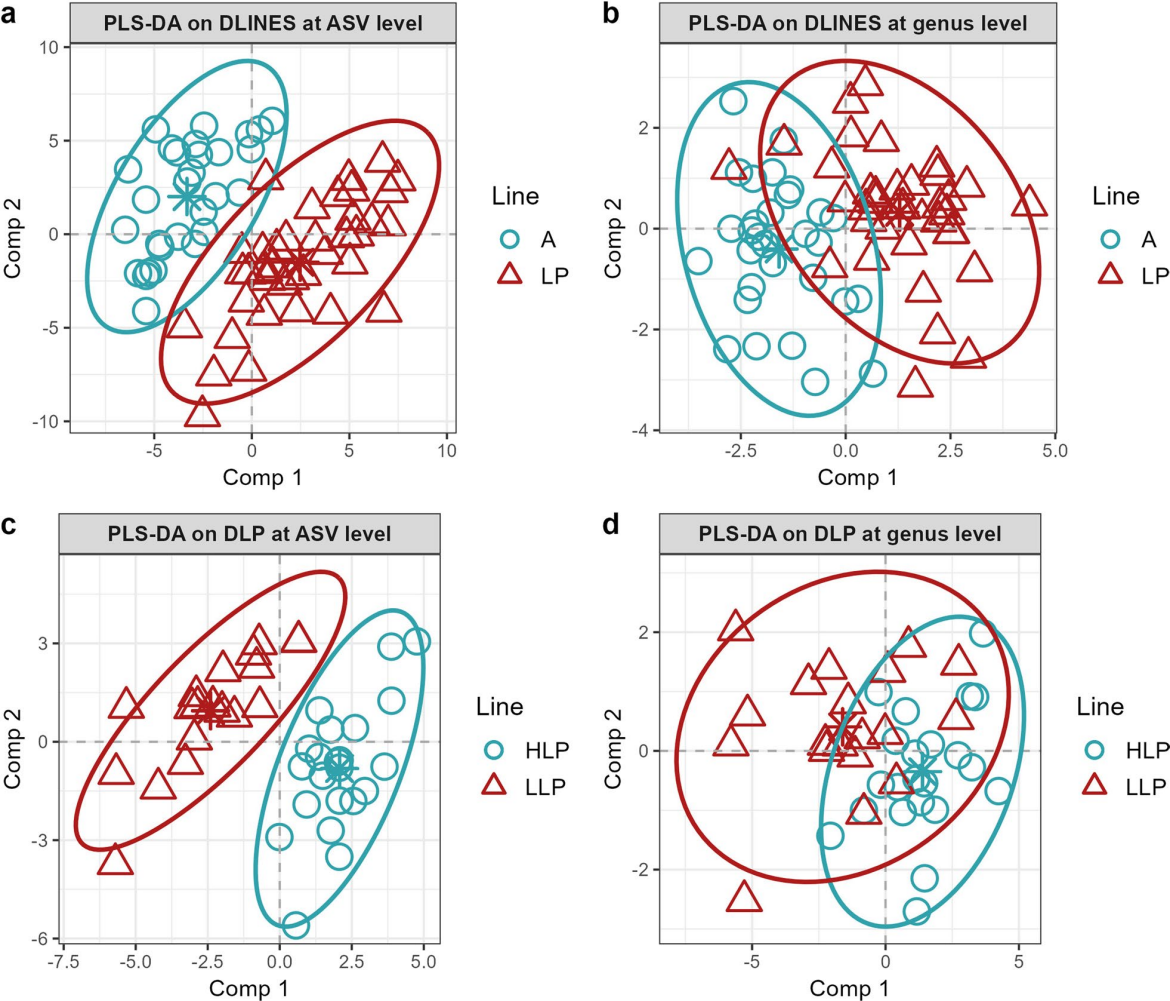
Individual plots of the results from the final partial least squares discriminant analysis (PLS-DA) models. **a** First and second components of the DLINES comparison (between lines A and LP) at the amplicon sequence variant (ASV) level. **b** First and second components of the DLINES comparison at the genus level. **c** First and second components of the DLP comparison (between LP does with two parities or less (LLP) and does with at least 15 parities (HLP) at the ASV level. **d** First and second components of PLSDA on the DLP comparison at the genus level

After ALR transformation, the final PLS-DA models identified 146 ASV and 22 genera that could discriminate between the A and LP lines (Table 1 and Fig. 3a and b). The final 146 ASV identified for the DLINES comparison were collapsed at the genus level, which allowed us to have taxonomic information about each ASV and to compare it with the results of the genus model (see Additional file 4: Table S1). Among the 22 genera identified in the DLINES comparison at the genus level, ASV that belonged to 10 of these genera were also identified in the model at the ASV level. The DLINES comparison at the ASV level showed a prediction performance (true positive rate) of 91% for line A, and 94% for line LP. At the genus level, the prediction performance was 85% for line A and 86% for line LP. The prediction performance of the permutation test for the DLINES comparison was 42% for line A and 58% for line LP at the ASV level and 48% for line A and 52% for line LP at the genus level. The permutation results suggest that the taxa identified in the PLS-DA did not randomly discriminate between groups.

**Table 1:** Partial least square discriminant analysis (PLS-DA) specifications using amplicon sequence variant (ASV) and genera taxa

Comparison	PLS-DA model	Number of variables	Number of components	Balance error rate (BER)	Standard deviation of BER
DLINES	ASV	146	3	0.09	0.03
	Genus	22	1	0.18	0.02
DLP	ASV	53	4	0.07	0.03
	Genus	20	1	0.28	0.02

DLINES: comparison between A and LP lines; DLP: comparison between LP does with two parities or less and those with at least 15 parities; BER: Balance error rate calculated as the average of the errors on each class

In the DLP comparison, the PLS-DA identified 53 ASV and 20 genera that allowed discrimination between the LLP and HLP groups (Table 1 and Fig. 3c and d). After collapsing the final 53 ASV to the genus level, of the 20 genera detected in the DLP comparison at the genus level, five were also identified in the model at the ASV level (see Additional file 4: Table S1). The confusion matrix for the DLP comparison resulted in prediction performances of 99% (HLP) and 94% (LLP) at the ASV level and of 82% (HLP) and 67% (LLP) at the genus level. The permutation test resulted in prediction performances of 53% for the HLP group and 47% for LLP at the ASV level and of 52% for HLP and 48% for LLP at the genus level. Due to the model's low prediction performance at the genus level (67% and 82%), the results and taxa identified in the DLP comparison at the genus level were excluded from further analyses and discussion.

The final PLS-DA models included 19 ASV that were common to the DLINES and DLP comparisons (see Additional file 4: Table S1). These ASV belonged to the genera *Christensenellaceae R-7 group*, *Colidextribacter*, *UCG-005, Bacteroides*, and *Tyzzerella*. The remaining ASV were not classified to the genus level, with some being classified only as bacteria, or to the order *Oscillospirales*, and others being classified to the family level, and belonging to *Lachnospiraceae*, *Eggerthellaceae*, *Atopobiaceae* and *Oscillospiraceae*.

### Differential abundance analysis

The differential abundance analysis revealed 105 ASV with significant differences in abundance between the A and LP lines. Of these, 26 ASV were more abundant in line LP with differences at least larger than 0.5 SD, while 21 ASV were more abundant in line A with differences larger than 0.5 SD (see Additional file 5: Table S2). These ASV were collapsed to the genus level and resulted in five genera that were more abundant in line LP and 12 genera that were more abundant in line A (Table 2). Differential abundance analysis at the genus level revealed 11 genera that exhibited differences in abundance larger than 0.5 SD between lines A and LP. Of these, nine were more abundant in line LP, including *Uncultured_Eubacteriaceae*, *Akkermansia*, *Parabacteroides* and others, while the genera *Ruminococcus* and *Lachnospiraceae UCG,001* were more abundant in line A (Table 2). There were some overlapping results between the ASV and genus models, with *Uncultured Eubacteriaceae*, *Akkermansia*, *Christensenellaceae R7 group,* and *Lachnospiraceae NK4B4 group* consistently more abundant in line LP at both levels. Similarly, *Ruminococcus* and *Lachnospiraceae UCG,001* were more abundant in line A at both levels.

**Table 2:** Genera with differential abundance greater than 0.5 standard deviations for the two comparisons

DLINES	Level	LP	A
	ASV	*Uncultured_Eubacteriaceae, Akkermansia, Christensenellaceae_R_7 group, Incertae_Sedis, Lachnospiraceae_NK4B4_group*	*[Eubacterium]_siraeum_group, Anaerofustis, Blautia, Clostridia_UCG_014, Colidextribacter*, *Lachnospiraceae_UCG,001, Flavonifractor, Methanobrevibacter, Oscillibacter, Ruminococcus, Subdoligranulum*, *UCG_005*
	Genus	*Uncultured_Eubacteriaceae, Akkermansia, Parabacteroides, Phascolarctobacterium, Enteroscipio, Christensenellaceae_R7_group, Shuttleworthia, Lachnospiraceae_NK4B4_group, Coprobacter*	*Ruminococcus*,* Lachnospiraceae_UCG_001*

DLP	Level	HLP	LLP
	ASV	*Uncultured_Eubacteriaceae, Subdoligranulum*, *Monoglobus*, *Sellimonas*	*Muribaculum*, *Blautia*, *Clostridia_vadinBB60_group*, *Clostridia_UCG_014*, *Colidextribacter*, *Alistipes*, *Bacteroides*, *Akkermansia*, *Butyricicoccus*, *Christensenellaceae_R_7_group*, *Lachnospiraceae_ND3007_group*, *Tyzzerella*, *UCG_005*

DLINES: comparison between lines A and LP; DLP: comparison between LP does with two parities or less (LLP) and those with at least 15 parities (HLP); ASV: amplicon sequence variance

In the differential abundance analysis of the DLP comparison, 37 ASV with differences in abundance larger than 0.5 SD between the LLP and HLP groups were identified (see Additional file 5: Table S2). Of these, six ASV were more abundant in the HLP group and the other 31 ASV were more abundant in the LLP group. These ASV resulted in four genera that were more abundant in the HLP group and 13 genera that were more abundant in the LLP group (Table 2).

Comparative analysis of the final taxa identified in both comparisons revealed that eight genera were common to the DLINES and DLP comparisons. Specifically, *Blautia*, *Clostridia UCG,014*, *Colidextribacter* and *UCG,005* were more abundant in line A (low longevity line) and in the LLP group (animals that died or were culled before their third parity). Conversely, *uncultured Eubacteriaceae* was more abundant in line LP (high longevity line) and in the HLP group (consisting of animals that had at least 15 parities). However, some genera showed opposite results, with *Akkermansia* and *Christensenellaceae R,7 group* being more abundant in line LP and in the LLP group, while *Subdoligranulum* was more abundant in line A and in the HLP group.

## Discussion

Among all the samples analysed, 11 bacterial phyla were detected in the gut microbiota of the rabbits. Consistent with previous findings [43], *Firmicutes* were by far the most abundant phylum. It has been shown that this phylum is classified as the most efficient for cellulose degradation and has a fundamental role in the digestion in rabbits [44]. *Actinobacteria* and *Bacteroidetes* were the second and third most abundant phyla, respectively. Xing et al. [45] also reported that *Actinobacteria* and *Bacteroidetes* were among the most abundant phyla in rabbits after *Firmicutes*. *Actinobacteria* and *Bacteroidetes* have been described as playing a major role in maintaining the gut barrier homeostasis [46]. At the family level, some of the most abundant bacterial families characterized in this study were *Lachnospiraceae* and *Ruminococcaceae*, which confirms a previous study by Cotozzolo et al. [47]. These families have been shown to play important roles in gut health [48]. Therefore, the observed microbiome composition of the most abundant bacteria was consistent with previous studies in rabbits [43, 45, 47].

The alpha and beta diversity indices showed differences in microbiome composition between lines A and LP. Greater alpha diversity was observed in line LP compared to line A. It should be noted that line LP has greater longevity than line A [24]. Hence, these findings are consistent with the idea that high microbial diversity is beneficial for coping with environmental stress and promotes a good health status [49, 50]. Animals with a more diverse microbial community are potentially better able to deal with pathogenic microbes [51]. The benefits of a high diversity have been linked to increased functional redundancies among the microbial community, which can contribute to a more stable metabolic state and better resilience to face variability in available feeding resources [52]. Likewise, in the DLP comparison (within line LP), we expected that the HLP group, with higher longevity, would have a higher diversity than the LLP group, but no differences in diversity were found.

Regarding the results from the PLS-DA, prediction performance based on the confusion matrix was high for the comparison between lines A and LP. At the ASV level, the results were 91% true positives for A and 94% for LP, and at the genus level 85% for A and 86% for LP. A slight decrease in prediction accuracy was observed when moving from the ASV model to the genus model. For the second comparison (DLP), prediction performance was also high at the ASV level, with 94% and 99% true positives for the LLP and HLP groups, respectively. Moving to the genus level, prediction performance dropped to 67% for the LLP and 82% for the HLP groups. More sample variability was noticeable, and LLP encompassed most of the HLP group (Fig. 3d). The drop in prediction performance from the ASV to the genus level can be attributed to the fact that the ASV are more specific than the genus level, as one genus can encompass multiple species, subspecies, and ASV, which reduces the discriminative ability of the PLS-DA genus model. However, because the drop in prediction performance was large in the DLP comparison (67% for LLP), the results and taxa identified in the DLP comparison at the genus level were discarded from further analyses, as they were considered unreliable because of the high error rate of predictions.

It is important to underline that the DLINES comparison involved two rabbit lines that are characterized by distinct genetic backgrounds. Line LP was founded based on longevity criteria [23], and it has consistently exhibited significantly greater longevity than line A [24]. The gut microbiota has been shown to be under genetic regulation [53, 54] and this may explain the observed differences in hologenomes [55]. In this experiment, both lines shared the same environmental and dietary conditions and, therefore, the observed differences are likely to be due to the genetics of the hosts. These different genetic backgrounds are the result of the underlying criteria used when the lines were founded but also of genetic drift. Thus, gut microbiome differences may or may not be fully associated with the differences in longevity between the two lines. However, differences observed between the HLP and LLP groups within line LP, which shared the same genetic background but differed only in terms of longevity, lend further support to the notion that the gut microbiota is a predictive factor for longevity in rabbits. If the specific taxa identified were found to be shared between both comparisons and/or to play a role in longevity and resilience in rabbits, this information could hold promise for improving these traits in rabbits.

A differential abundance analysis was performed using Bayesian statistics to detect the most reliable taxa and to determine in which lines (A and LP) and groups (LLP and HLP) they were more abundant. The genus *Uncultured Eubacteriaceae* was one of the few relevant genera that was identified in the DLINES and DLP comparisons. This genus was found in great abundance in the groups with longer longevity, i.e., in line LP and group HLP. In a study comparing two rabbit lines, i.e. Sichuan White and New Zealand rabbits, a higher abundance of *Uncultured Eubacteriaceae* was found in the Sichuan White rabbit group, which also exhibited a significantly higher survival rate (p < 0.05) compared to the New Zealand rabbit group [56]. Line A in this study was founded from New Zealand rabbits and had a lower survival rate than line LP [24], supporting the possible importance of the *Uncultured Eubacteriaceae* in modulating longevity. *Blautia* and *Colidextribacter* were found to be more abundant in line A and in group LLP. Similarly, *Blautia* was more abundant in the New Zealand rabbit group that is characterized with a lower survival rate [56]. Regarding *Colidextribacter*, a study conducted in cattle found a significant increase in the genus *Colidextribacter* in milk samples taken from cows with mastitis [57]. It should be noted that mastitis is among the main reproduction-related causes that lead to culling in rabbits [58]. *Akkermansia* and *Christensenellaceae R-7 group* were also identified in both comparisons. In the DLINES comparison, they were more abundant in line LP. Similarly, the Sichuan White rabbits’ line, which had a higher survival rate, was also found to have a greater abundance of both *Akkermansia* and *Christensenellaceae R-7 group* compared to the New Zealand rabbit line [56]. The *Christensenellaceae* family is one of the most heritable families in gut microbiota, with its abundance having a heritability of 0.42 (95% CI = 0.25–0.48) [59]. The differences in genetic make-up between the LP and A lines may explain the difference in abundance of the *Christensenellaceae R-7* group between these two lines, thus highlighting the role of the host genome in influencing longevity, including through its impact on the microbiome. Notably, some of the findings regarding differences in the abundance of genera, both between the rabbit lines and between animals within the same line, are consistent with genera that were associated with resilience and host genetics in a previous rabbit study [35]. The genus *Parabacteroides* was found to be more abundant in line LP (DLINES comparison), which is consistent with the findings of Casto-Rebollo [35] who observed a higher abundance of *Parabacteroides *sp. in a rabbit line with greater resilience. Similarly, *Flavonifractor* and *Ruminococcus* were more abundant in line A, and *Muribaculum* was more abundant in group LLP, which mirrors the findings of Casto-Rebollo [35], who showed that these genera were more abundant in non-resilient rabbit lines.

An important result from the abundance analysis, is the shared ASV and genera identified for the DLINES and DLP comparisons, including *Uncultured Eubacteriaceae*, *Blautia*, *Colidextribacter*, *Akkermansia* and *Christensenellaceae R-7 group.* These taxa may be related to longevity since they discriminated between lines A and LP, which have different genetic backgrounds, and between groups LLP and HLP within the same line. Moreover, in the literature, these taxa have been directly associated with longevity in rabbits, or with other related traits, such as resilience. If there is a direct association or a possible influence of these taxa on longevity and/or resilience, their abundance could be used as a selection criterion for these traits and included in their prediction model [21, 22]. An interesting alternative is to create a probiotic to modify the rabbit’s gut microbiota.

## Conclusions

Our results support that the gut microbiome differs between the two rabbit maternal lines LP and A, with line LP having a superior longevity compared to line A. We found differences in the gut microbiome between animals with different longevities within line LP, LLP (died or culled with 2 parities or less) and HLP (minimum of 15 parities). Line LP had a higher alpha diversity than line A, which is consistent with the prevailing thinking that a more diverse microbiota is associated with greater resilience and longevity. In addition, PLS-DA identified taxa that discriminate between groups with different longevities. Although further studies are needed to validate these results, in the literature, some of these genera were found to be regulated by the host genetics, thus, could be used as an alternative criterion to select for longevity. An alternative would be to develop a probiotic to deliberately modify the gut microbiota of rabbits and increase their longevity.

### Supplementary Information

**Additional file 1**: **Text S1.** The Reference ASV used for ALR transformation in the DLINES and DLP comparisons.

**Additional file 2:** **Text S2.** The sequences of all ASV identified with their ID.

**Additional file 3:** **Figure S1.** Alpha diversity boxplots of Pielou’s evenness index (p-value = 0.17) computed at the amplicon sequence variant (ASV) level for lines A (standard commercial maternal line) and LP (maternal line founded using longevity criteria. Boxplots of alpha diversity indices with non-significant differences between the groups according to the Kruskal–Wallis test. **Figure S2.** Alpha diversity boxplots of observed diversity index (p-value = 0.15) computed at the amplicon sequence variant (ASV) level for the DLP comparison: between LP does with two parities or less (LLP) and those with at least 15 parities (HLP). **Figure S3.** Alpha diversity boxplots of Pielou’s evenness diversity index (p-value = 0.31) computed at the amplicon sequence variant (ASV) level for the DLP comparison: between LP does with two parities or less (LLP) and those with at least 15 parities (HLP). **Figure S4.** Alpha diversity boxplots of the Shannon diversity index (p-value = 0.08) computed at the amplicon sequence variant (ASV) level for the DLP comparison: between LP does with two parities or less (LLP) and those with at least 15 parities (HLP).

**Additional file 4: Table S1.** The ASV and genera identified in the final DLINES and DLP PLS-DA models and their taxonomic information. Table S1 includes three sheets: in the first sheet, are provided from left to right; the ID of the ASV identified in the final DLINES PLS-DA model at the ASV level, the taxonomic information of each ASV, and the genera identified in the final DLINES PLS-DA model at the genus level; in the second sheet, are provided the ID of the ASV identified in the final DLP PLS-DA model at the ASV level, the taxonomic information of each ASV, and the genera identified in the final DLP PLS-DA model at the genus level; and the third sheet contains the ASV ID found in common between the two comparison DLINES and DLP in their final PLS-DA model at the ASV level, and their taxonomic information.

**Additional file 5:** **Table S2.** Results of the differential abundance analysis using Bayesian statistical models. Table S2 includes three sheets, the first sheet shows the results of the Bayesian statistical analysis of the DLINES comparison at the ASV level; the second sheet shows, the results of the Bayesian statistical analysis of the DLINES comparison at the genus level; and the third sheet shows the results of the Bayesian statistical analysis of the DLP comparison at the ASV level. In each sheet (from left to right) the columns are, the ASV ID and the taxonomic information (or the genus name), posterior mean of the differences among the two groups of the comparison (line A and line LP for the DLINES comparison, and between LLP and HLP groups for the DLP comparison) (meanDiff), the probability of the difference being larger (if the difference is positive) or smaller (if negative) than 0 (P0), the highest posterior interval density of 95% (HPD95), and the cumulative PEP.

## Data Availability

The data for this study have been deposited in the European Nucleotide Archive (ENA) at EMBL-EBI under accession number PRJEB71513 (https://www.ebi.ac.uk/ena/browser/view/PRJEB71513).
