# Supplementary material for: Microbiome composition as a potential predictor of longevity in rabbits
Source: Genet Sel Evol. 2024 Apr 2;56:25. doi: 10.1186/s12711-024-00895-6 (PMC10986140; doi:10.1186/s12711-024-00895-6)
Supplement: Supplementary file 3 — Additional file 3: Figure S1. Alpha diversity boxplots of Pielou’s evenness index (p-value = 0.17) computed at the amplicon sequence variant (ASV) level for lines A (standard commercial maternal line) and LP (maternal line founded using longevity criteria. Boxplots of alpha diversity indices with non-significant differences between the groups according to the Kruskal–Wallis test. Figure S2. Alpha diversity boxplots of observed diversity index (p-value = 0.15) computed at the amplicon sequence variant (ASV) level for the DLP comparison: between LP does with two parities or less (LLP) and those with at least 15 parities (HLP). Figure S3. Alpha diversity boxplots of Pielou’s evenness diversity index (p-value = 0.31) computed at the amplicon sequence variant (ASV) level for the DLP comparison: between LP does with two parities or less (LLP) and those with at least 15 parities (HLP). Figure S4. Alpha diversity boxplots of the Shannon diversity index (p-value = 0.08) computed at the amplicon sequence variant (ASV) level for the DLP comparison: between LP does with two parities or less (LLP) and those with at least 15 parities (HLP). [file 12711_2024_895_MOESM3_ESM.docx]

**Alpha diversity Boxplots**

**
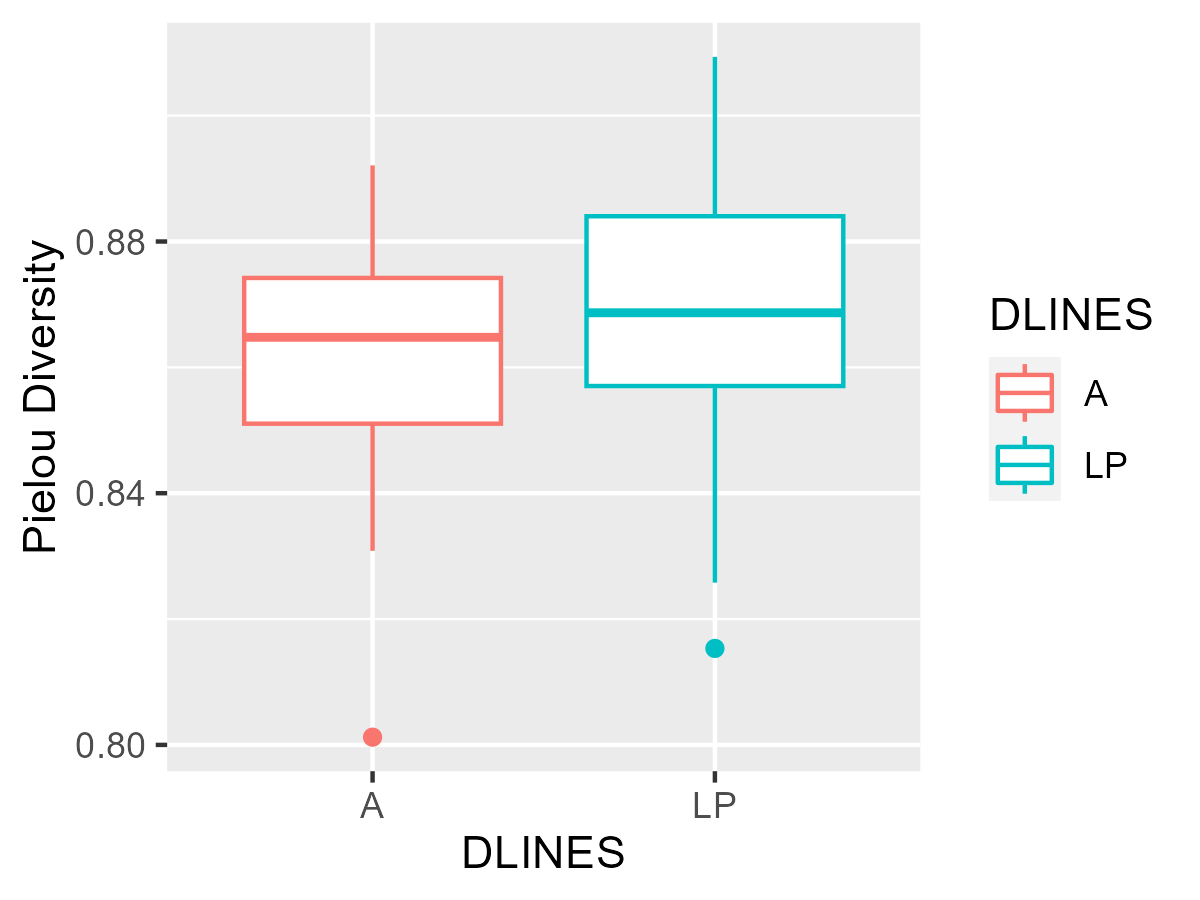
**

**Figure S1.** Alpha diversity boxplots of Pielou Evenness index (p-value = 0.17) computed at amplicon sequence variant (ASV) level for line A (standard commercial maternal line) and LP (maternal line founded using longevity criteria).

**Pielou Evenness**


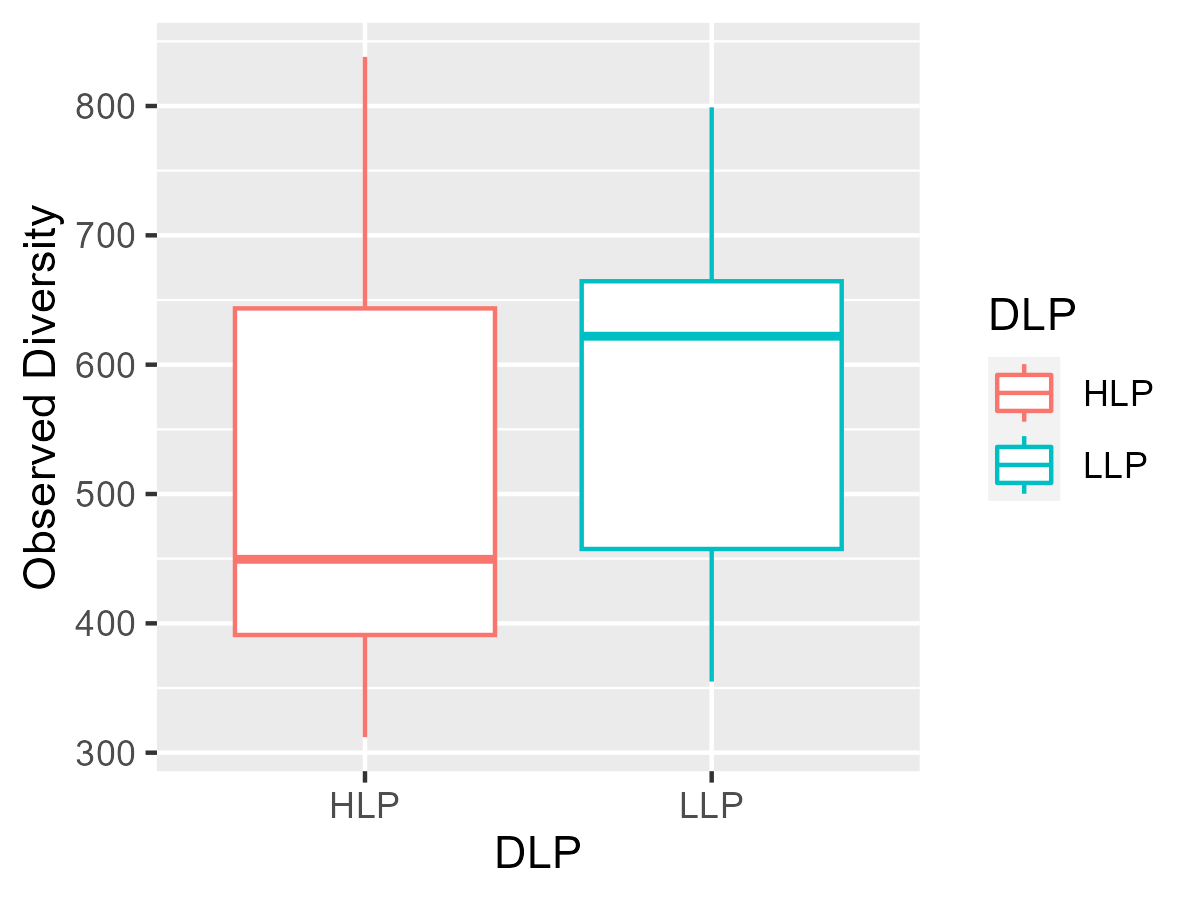


**Figure *S2*.** Alpha diversity boxplots of Observed diversity index (p-value = 0.15) computed at amplicon sequence variant (ASV) level for DLP comparison: between LP does with two parities or less (LLP) and those with at least 15 parities (HLP).

**Pielou Evenness**


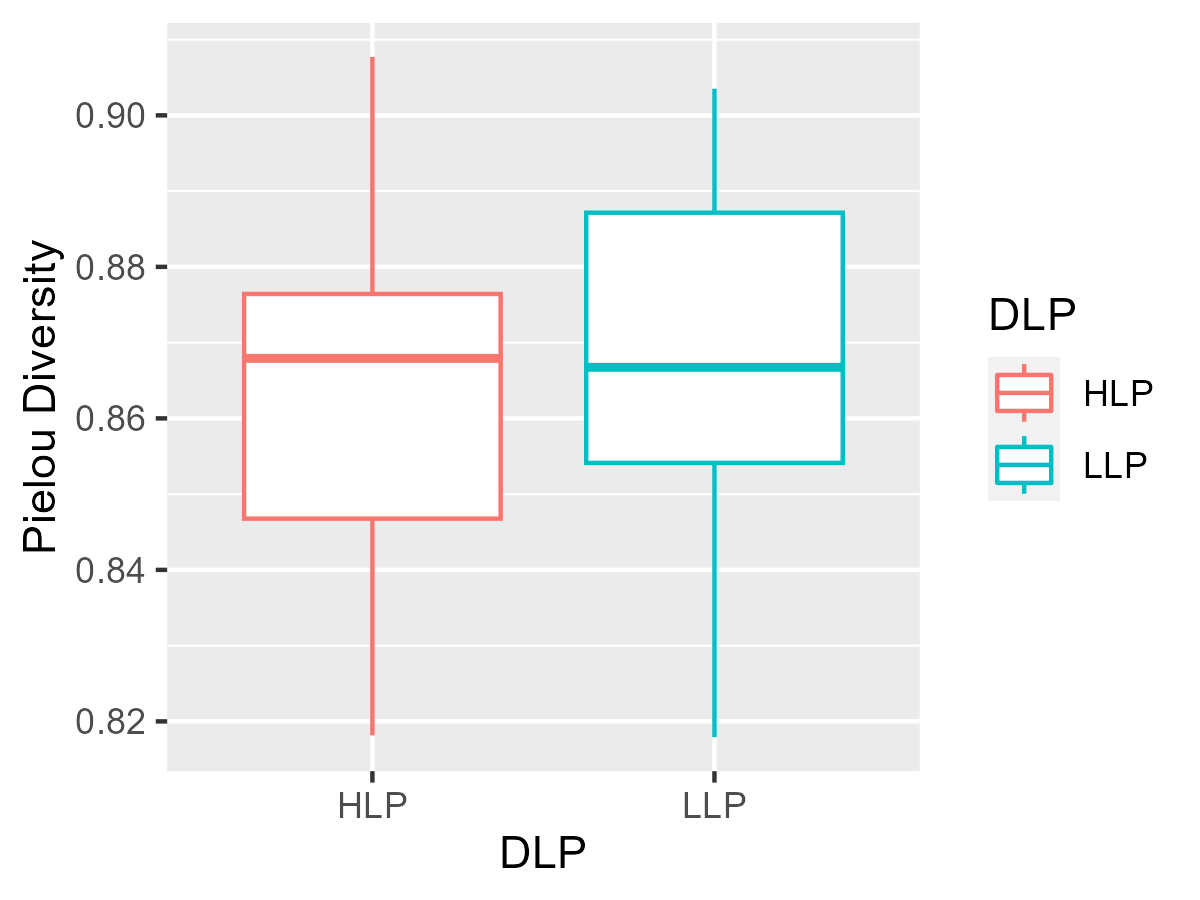


**Figure *S3*.** Alpha diversity boxplots of Pielou Evenness diversity index (p-value = 0.31) computed at amplicon sequence variant (ASV) level for DLP comparison: between LP does with two parities or less (LLP) and those with at least 15 parities (HLP).

**Pielou Evenness**


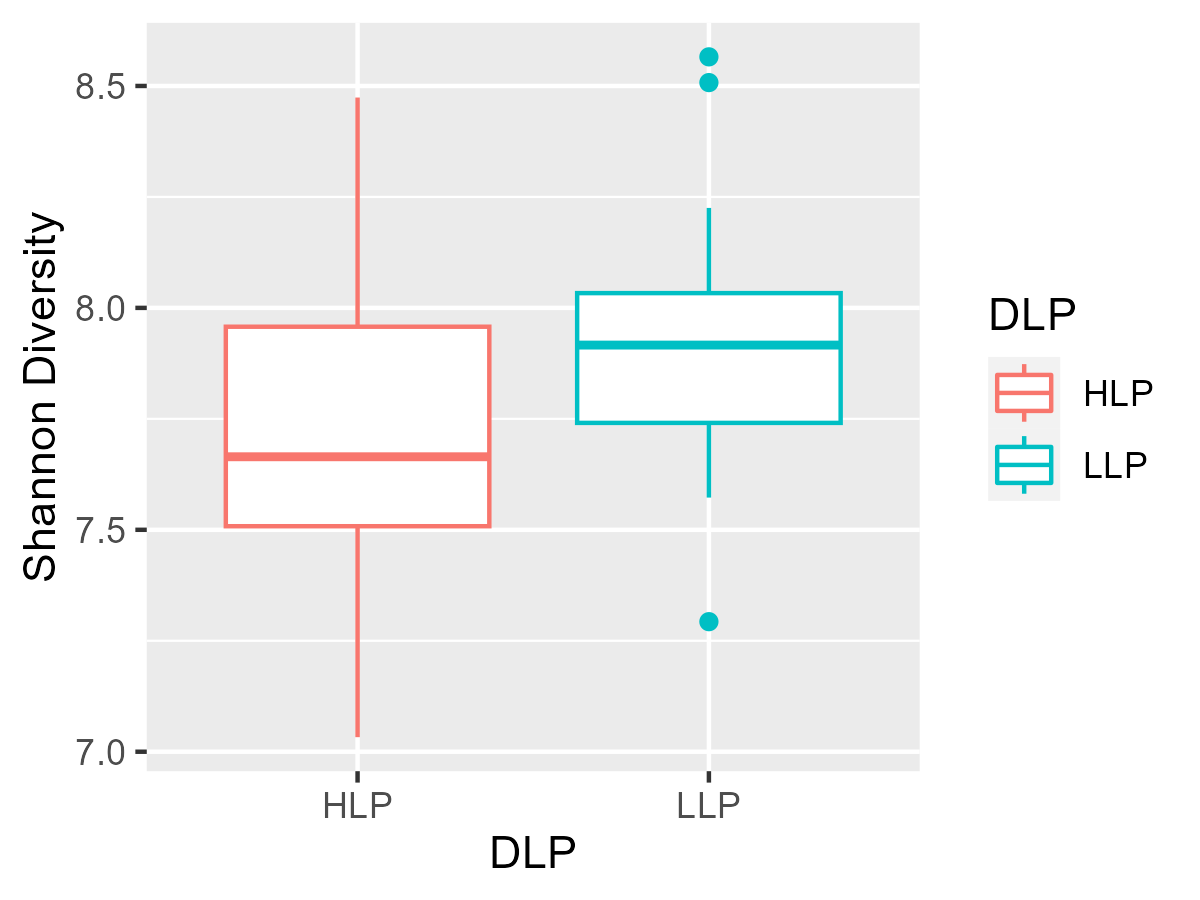


**Figure *S4*.** Alpha diversity boxplots of Shannon diversity index (p-value = 0.08) computed at amplicon sequence variant (ASV) level for DLP comparison: between LP does with two parities or less (LLP) and those with at least 15 parities (HLP).

**Pielou Evenness**
